# Validation of SeLECT score and its modification for predicting unprovoked epileptic seizures in patients after ischemic stroke

**DOI:** 10.1097/MD.0000000000047067

**Published:** 2026-01-23

**Authors:** Petr Janský, Tereza Šrámková, Jakob Reichl, Milan S. Vosko, Lucie Šťovíčková, Anna Olšerová, Kateřina Benešová, Silvia Kmetonyová, Jaroslava Paulasová-Schwabová, Jakub Otáhal, Petr Marusič, Milan R. Vosko, Aleš Tomek

**Affiliations:** a Department of Neurology, Second Faculty of Medicine, Charles University and Motol University Hospital, Prague, Czech Republic; b Department of Neurology 2, KeplerUniversitätsklinikum, Med Campus III, Linz, Austria; c Department of Neurosurgery, KeplerUniversitätsklinikum, Med Campus III, Linz, Austria; d Department of Pathophysiology, Second Faculty of Medicine, Charles University and Motol University Hospital Motol, Prague, Czech Republic.

**Keywords:** biomarker, ischemic stroke, SeLECT score, vascular epilepsy

## Abstract

Vascular epilepsy represents a significant late complication of ischemic stroke. SeLECT score is an important prediction model for late poststroke seizures. The study aimed to validate the ability of the SeLECT score, its parameters, and other selected biomarkers to predict the occurrence of unprovoked epileptic seizures in patients after ischemic stroke. Retrospective analysis of consecutive supratentorial ischemic stroke survivors with a negative history of epilepsy, admitted to 2 major comprehensive stroke centers in the Czech Republic and Austria in 1 year period. The follow-up information was collected from available medical documentation, using a structured telephone questionnaire and patient visits. Three hundred fifteen patients were included (59% men, median age 69 years, median National Institutes of Health Stroke Scale [NIHSS] 4, intravenous thrombolysis was administered to 29.2%, mechanical thrombectomy was done in 6.3%). Unprovoked epileptic seizures occurred in 24 patients (7.6%), and the median follow-up period was 3.3 years. Vascular epilepsy was significantly associated with cortical localization of the ischemic lesion (hazard ratio [HR] 3.324; 95% confidence intervals [CI] 1.432–7.714; *P* = .005), higher NIHSS at discharge (HR 1.1; 95% CI 1.017–1.191; *P* = .018), and a history of prior stroke (HR 4.098; 95% CI 1.86–9.03; *P* < .001). Validation of the SeLECT score confirmed its predictive capabilities (area under the receiver operating characteristic curve 0.668; *P* = .005), and modifications incorporating NIHSS at discharge improved its performance (area under the receiver operating characteristic curve 0.715; *P* = .001).

## 1. Introduction

Unprovoked epileptic seizures are among the significant late complications of ischemic stroke.^[1]^ Ischemic stroke accounts for nearly 50% of newly diagnosed epileptic seizures in patients over 60 years old^[2]^ and is responsible for up to 11% of adult epilepsy cases overall.^[3]^ According to a nationwide Swedish study, the occurrence of vascular epilepsy is associated with a significantly higher risk of overall mortality (hazard ratio [HR] 1.68; 95% confidence intervals [CI] 1.25–1.53), even after adjusting for factors such as age, comorbidities, and stroke severity.^[4]^ In addition to increased mortality, worsening functional disability was documented in patients with recurrent epileptic seizures due to suboptimally controlled vascular epilepsy (OR 3.26; *P* = .01), depending on the frequency of seizure occurrence.^[5]^

The diagnosis of structural epilepsy following a stroke (poststroke epilepsy and vascular epilepsy) can be established with the occurrence of a single unprovoked epileptic seizure after a stroke. It has been reported that the probability of recurrence after the first unprovoked seizure following a stroke is 71.5% (95% CI 59.7–81.9),^[6]^ which exceeds the 60% threshold for the likelihood of seizure recurrence within the next 10 years required for an epilepsy diagnosis.^[7]^ In patients after stroke, a late (unprovoked) seizure is arbitrarily defined as one occurring more than 7 days after a stroke, whereas seizures occurring within 7 days are classified as early (acute symptomatic seizures).^[8]^ The distinction between acute symptomatic seizures and vascular epilepsy also has therapeutic implications, as patients with acute symptomatic seizures are typically not indicated for long-term antiseizure medication, whereas treatment is recommended for those with a first unprovoked seizure after a stroke.^[8]^ Treatment decisions must consider gender, drug interaction and age-related pharmacokinetics, comorbidities, and polypharmacy. Levetiracetam and lamotrigine are generally preferred due to tolerability and low interaction potential.

Reported seizure incidence following stroke varies in the literature from 2% to 20%, with higher rates observed in cases of hemorrhagic stroke and subarachnoid hemorrhage. A large recent Danish national cohort study found that the cumulative incidence of epilepsy following ischemic stroke was 3% (95% CI 2.9–3.2) at 2 years and 8.6% (95% CI 8.0–9.2) following intracerebral hemorrhage. At 10 years, the incidence was 5.1% after ischemic stroke and 12.5% after intracerebral hemorrhage.^[9]^

The development of acute symptomatic seizures following ischemic stroke is mainly influenced by increased extracellular potassium and glutamate concentrations due to ischemic neuronal damage, leading to ion channel dysfunction, blood–brain barrier disruption, and metabolic imbalance, ultimately resulting in heightened neuronal excitability.^[10]^ In contrast, the development of vascular epilepsy after ischemic stroke involves multiple factors and mechanisms related to structural and functional remodeling of the damaged brain tissue, leading to persistent changes in neuronal excitability and connectivity. This reorganization transforms normal brain tissue into epileptic tissue with an endogenous capacity to generate seizures. Mechanisms contributing to epileptogenesis include gliotic scar formation, chronic inflammation, angiogenesis, neurodegeneration, changes in synaptic plasticity, and alterations in ion channel expression.^[11]^

The epileptogenic process evolves over time following a stroke, and different epileptogenic mechanisms may be linked to different biomarkers, which could not only predict epilepsy development but also allow for staging of the epileptogenic process.^[12]^ Several risk factors and biomarkers have been identified for estimating the risk of vascular epilepsy, including clinical factors, imaging biomarkers, biochemical markers, genetic markers, and electroencephalographic markers. Clinical risk factors include stroke severity, the presence of acute symptomatic seizures, younger age, dementia, diabetes mellitus, arterial hypertension, and dyslipidemia.^[13]^

Among the most significant imaging risk factors and biomarkers, most commonly detected through MRI, are hemorrhagic transformation of the ischemic lesion,^[14]^ lesion volume,^[4]^ cortical lesion location, involvement of the middle cerebral artery (MCA) territory,^[15]^ leukoaraiosis, cerebral microbleeds, and cortical superficial siderosis.^[16]^

The effort to integrate individual risk factors and enhance their predictive value has led to the development of prognostic models. The most robust predictive tool for unprovoked seizures in stroke patients is the SeLECT score,^[15]^ introduced in 2018. The name SeLECT is an acronym for 5 risk factors included in its calculation: stroke severity, presence of large-artery disease, occurrence of acute symptomatic seizures in connection with the initial stroke, cortical lesion location, and involvement of the MCA territory. Each risk factor is assigned points, and their sum determines the final score. A higher score correlates with a greater risk of unprovoked seizures. A score of zero points corresponds to a 1.3% risk (95% CI 0.7–1.8) of developing vascular epilepsy over 5 years, whereas a score of 9 points corresponds to a 63% risk (95% CI 62–93).

Recently, several modifications of the SeLECT score have emerged to further enhance its predictive capabilities. In 2023, SeLECT was updated to SeLECT 2.0, which differentiates acute symptomatic seizures into separately scored brief seizures and status epilepticus, making it more effective in identifying patients at high risk of vascular epilepsy.^[17]^ Early electrographic biomarkers have also been incorporated into the SeLECT-EEG score.^[18]^

## 2. Aim of the study

The aim of the study was to validate the ability of the SeLECT score, its parameters, and other selected biomarkers to predict the occurrence of unprovoked epileptic seizures in patients after ischemic stroke.

We hypothesized that the cumulative risk of unprovoked epileptic seizures over time after ischemic stroke increases in correlation with the SeLECT score values.

We further hypothesized that the cumulative risk of unprovoked epileptic seizures over time after ischemic stroke increases based on the individual parameters used in the SeLECT score calculation, including stroke severity, large-artery disease, the occurrence of acute symptomatic epileptic seizures in connection with the initial ischemic stroke, cortical lesion localization, and involvement of the MCA territory.

Another hypothesis was that a modified SeLECT score, incorporating the National Institutes of Health Stroke Scale (NIHSS) at discharge instead of the initial NIHSS, would have greater sensitivity and specificity in predicting unprovoked epileptic seizures after ischemic stroke compared to the original SeLECT score.

## 3. Methods

### 3.1. Study population

The study included adult patients who had suffered an ischemic stroke and were consecutively hospitalized in 2015 at 2 comprehensive cerebrovascular centers in the Czech Republic (Motol University Hospital, Prague) and Austria (Kepler University Hospital, Linz). In both institutions, a list of hospitalizations coded under the International Classification of Diseases for ischemic stroke (I63.x) was obtained from the hospital information system. From this list, individual patients who had experienced an acute or subacute ischemic stroke were manually identified. Patients with a history of epilepsy or those taking antiseizure medication were excluded, except for those using gabapentin, pregabalin, or benzodiazepines for non-epileptic indications. Additionally, patients with isolated infratentorial ischemic stroke and those who died within 2 weeks of the stroke were excluded.

After removing incorrectly coded, duplicate, and otherwise invalid hospitalizations, 510 patients hospitalized for ischemic stroke in 2015 were identified in the Czech cohort, and 403 in the Austrian cohort. In the Czech cohort, 190 patients did not meet the inclusion criteria: 128 due to the presence of an isolated infratentorial lesion, 14 due to a history or current treatment of epilepsy, and 48 due to early death within 2 weeks of the stroke. In the Austrian cohort, 108 patients did not meet the inclusion criteria: 80 due to an isolated infratentorial lesion, 6 due to a history or current treatment of epilepsy, and 22 due to early death within 2 weeks of the stroke. From the 320 patients in the Czech cohort who met the inclusion criteria, 102 were excluded due to loss to follow-up (patients who declined the questionnaire, had died, or had no available contact information and no sufficient medical documentation). Similarly, in the Austrian cohort, 198 patients out of the 295 meeting the inclusion criteria were excluded for the same reasons. A total of 315 patients were included in the study, with 218 from the Czech Republic and 97 from Austria. The same study population was used in our previous analysis aimed at describing the occurrence and risk factors of unprovoked seizures after ischemic stroke.^[19]^

### 3.2. Patient follow-up and telephone survey

To monitor the health status of patients, a structured telephone interview with the patient or a close relative (household member) was used as the primary source of information. The interview was based on a modified version of an epilepsy questionnaire used in clinical practice at the Neurology Department of Motol University Hospital. The questionnaire also included questions about recurrent ischemic stroke and transient ischemic attacks (TIAs). Both the Czech and German versions of the questionnaire were administered by medical students who were specifically trained for this purpose. The telephone interviews were conducted from April 2018 to October 2019. In cases where an epileptic seizure was suspected or there were uncertainties during the interview, the patient was re-contacted by a physician. Patients whose questionnaire results suggested epilepsy were invited for an outpatient neurological examination. In borderline cases – especially for patients with significant neurological deficits after an ischemic stroke that could make seizure identification difficult – the diagnosis was assessed by a broader medical team. To rule out acute symptomatic seizures in cases of possible recurrent ischemic stroke, imaging and laboratory tests were performed when there was reasonable suspicion. Patients who could not be reached by phone were sent the questionnaire by mail. If the questionnaire could not be delivered (e.g., the patient had died, refused to participate, could not be reached by phone despite 3 attempts on different days and times, or did not respond to the mailed questionnaire), their general practitioner was contacted for further information. In some cases, the hospital’s medical records were reviewed. Medical documentation that did not mention seizures was considered a sufficient source of information if it came from a neurology department at least 1 year after the ischemic stroke. Patients whose seizures could not be verified by any of the these methods were excluded from the study. The follow-up data collection in the Austrian cohort was more limited; besides telephone interviews, only hospital medical records were available. Mailing questionnaires or contacting general practitioners was not possible in Austria.

### 3.3. Data collection and statistical analysis

The data regarding the initial hospitalization were retrieved from hospital information systems. The collected data covered multiple categories, including ischemic stroke characteristics, epilepsy risk factors, vascular risk factors, medication, laboratory values, and the occurrence of acute symptomatic seizures. Particular attention was given to the parameters of the SeLECT score (severity of ischemic stroke, large artery disease, presence of acute symptomatic seizures related to the initial ischemic stroke, cortical lesion location, and MCA involvement). All data were stored in the REDCap database system (Research Electronic Data Capture, Vanderbilt University, Nashville) under anonymized identifiers.

Statistical analysis was performed using SPSS Statistics 25 (IBM, Armonk). Descriptive statistics were used to characterize all patients and subgroups based on the development of vascular epilepsy and hospital site in the retrospective study. Statistical significance between subgroups with and without vascular epilepsy was assessed using the chi-square test for categorical variables and the Mann–Whitney *U* test or Student *t* test for continuous variables. Continuous variables were presented as median with interquartile range (IQR) or mean with standard deviation, depending on data distribution. The IQR was defined as the difference between the 75th and 25th percentiles (Q1–Q3). Categorical variables were presented as absolute frequencies and percentages. The SeLECT score was calculated both in its original form^[15]^ and in a modified version using NIHSS scores at discharge instead of admission values. To compare the distribution of the SeLECT score between patients with and without seizures, the Independent Samples Proportions test with Wald test for the null hypothesis was used. Associations between various covariates (selected clinical, radiological, and laboratory parameters, including the SeLECT score) and the primary outcome (occurrence of vascular epilepsy) were analyzed using survival analysis methods. The Cox proportional hazards model was used to calculate HRs with 95% CIs. Additionally, Kaplan–Meier survival curves were generated, and log-rank tests were used to compare survival distributions. The annual risk of vascular epilepsy was assessed across different SeLECT score subgroups. The discriminative ability of SeLECT as a diagnostic test was evaluated using ROC analysis, including the sensitivity and specificity of different score values. The optimal cutoff value for the models using admission and discharge NIHSS scores was determined using Youden index. Schoenfeld residuals were calculated for all models to check for significant deviations from the proportional hazards assumption. A *P*-value < .05 was considered statistically significant.

### 3.4. Definitions, scales, and classifications used

Vascular epilepsy was diagnosed if at least 1 unprovoked seizure occurred at least 7 days after an ischemic stroke. Seizures were classified according to the International League Against Epilepsy 2017 criteria.^[20]^ Stroke etiology was determined using the Trial of Org 10172 in Acute Stroke Treatment classification.^[21]^ Stroke severity was assessed using the National Institute of Health Stroke Scale (NIHSS).^[22]^

## 4. Results

### 4.1. Characteristics of the patient cohort and recorded seizures

The overall cohort (Table 1) consisted of 59% male patients, with a median age of 69 years (IQR 17). The median NIHSS score was 4 (IQR 4). Intravenous thrombolysis was administered in 29% of patients, and mechanical thrombectomy was performed in 6%. The most common etiology of ischemic stroke was cardioembolic (32%), followed by large artery disease (30%), cryptogenic etiology (23%), small vessel disease (8%), and other determined causes (8%). NIHSS ≤ 3 was observed in 49% of patients, NIHSS 4 to 10 in 40%, and NIHSS ≥ 10 in 11% of patients. Cortical ischemic lesions were present in 41% of patients, and acute symptomatic seizures associated with ischemic stroke were recorded in 4% of cases. The MCA territory was affected in 84% of cases.

**Table 1:** Patient characteristics.

	All patients (n = 315)	No seizure n = 291	Vascular epilepsy n = 24	*P < .05
Follow-up time – days, median (IQR)	1206 (377)	1194 (377)	1281 (673)	.862
Age – years, median (IQR)	69 (17)	69 (17)	67.5 (18)	.653
Male – number (%)	186 (59%)	169 (58%)	17 (71%)	.222
NIHSS admission value (median, IQR)	4 (4)	4 (4)	6 (5)	.062
NIHSS discharge value (median, IQR)	1 (3)	1 (3)	2 (6)	.079
*Severity of ischemic stroke at discharge (value missing in 46 patients*)				
NIHSS ≤ 3	224 (83%)	209 (85%)	15 (65%)	.035*
NIHSS 4–10	35 (13%)	29 (12%)	6 (26%)	.095
NIHSS ≥ 11	10 (4%)	8 (3%)	2 (9%)	.207
Intravenous thrombolysis – number (%)	92 (29%)	84 (29 %)	8 (33%)	.644
Mechanical thrombectomy – number (%)	20 (6%)	18 (6%)	2 (8%)	.657
*SeLECT score parameters – number (%*)				
Severity of ischemic stroke on admission				
NIHSS ≤ 3	155 (49%)	147 (51%)	8 (33%)	.106
NIHSS 4–10	127 (40%)	113 (39%)	14 (58%)	.061
NIHSS ≥ 11	33 (11%)	31 (11%)	2 (8%)	>.999
Large artery disease	93 (30%)	82 (28%)	11 (46%)	.068
Cortical localization of lesion	129 (41%)	112 (39%)	17 (71%)	.002*
Acute symptomatic seizure	12 (4%)	10 (3%)	2 (8%)	.23
Affected territory of MCA	264 (84%)	243 (84%)	21 (88%)	.778
SeLECT score – median (IQR)	2 (3)	2 (3)	4 (3)	.002*
*Events during follow-up – number (%*)				
Ischemic stroke	35 (11%)	31 (11%)	4 (17%)	.322
Transitory ischemic attack	6 (2%)	6 (2%)	0 (0%)	>.999
Death	39 (12%)	37 (13%)	2 (8%)	.751
None of the above	242 (77%)	223 (77%)	19 (79%)	.777

IQR = interquartile range, MCA = middle cerebral artery, NIHSS = National Institutes of Health Stroke Scale.

During the follow-up period (median duration 1206 days; IQR 377), unprovoked seizures were detected in 7.6% of patients. A recurrent ischemic stroke occurred in 11.1% of patients, while 1.9% experienced a TIA. During the observation period, 12.4% of patients died.

Of the 24 patients with confirmed unprovoked seizures, 15 cases (62.5%) were documented in hospital records, while 9 cases (37.5%) were identified through telephone surveys and subsequently confirmed by clinical examination. An additional 10 cases were initially misclassified as seizures by the telephone questionnaire but were ruled out upon clinical evaluation, with the presenting symptoms most often attributable to spasticity or other conditions, such as mild traumatic brain injury, carpal tunnel syndrome, or neuropathy.

The median time to the first unprovoked seizure was 407 days (IQR 403). The most frequent seizure type was focal seizure evolving into bilateral tonic-clonic seizures (11 patients; 46%). Among focal seizures without motor manifestations (7 patients; 30%), sensory and cognitive seizures were observed. Among focal motor seizures (6 patients; 25%), clonic, atonic, and myoclonic seizures were recorded. No cases of status epilepticus, including nonconvulsive status epilepticus, were observed.

All patients who experienced seizures were prescribed antiseizure medication, with levetiracetam being the most commonly used drug (21 patients; 88%), followed by lamotrigine (2 patients; 8%) and topiramate (1 patient; 4%). Two patients required a change in antiseizure treatment during the follow-up period.

### 4.2. SeLECT score and additional risk factors for vascular epilepsy

The median SeLECT score (Table 1) in patients with vascular epilepsy was 4 (IQR 3), whereas in patients without unprovoked seizures, the median SeLECT score was 2 (IQR 3). This difference was found to be statistically significant (*P* = .002). Patients with unprovoked seizures had a significantly higher occurrence of cortical lesion localization (70.8% vs 38.5%; *P* = .002). This group also showed higher NIHSS scores at admission, but neither the difference in median NIHSS scores (median 6 vs 4; *P* = .062) nor the proportion of patients in defined NIHSS ranges was statistically significant. A more pronounced difference between the groups was observed when evaluating NIHSS at discharge, where the proportion of patients with NIHSS ≤ 3 was significantly lower in those with vascular epilepsy (65.2% vs 85%; *P* = .035). Other SeLECT score risk factors and basic clinical characteristics did not differ significantly between the 2 groups. The number of ischemic strokes, TIAs, or deaths during the follow-up period did not significantly differ between patients with vascular epilepsy and those without unprovoked seizures. The median SeLECT score in patients with recurrent stroke (2; IQR 1.5–4; n = 35) was not significantly different from that in patients without recurrence (2.5; IQR 1.75–4; *P* = .13).

Univariate Cox regression analysis of predictors associated with unprovoked seizure occurrence during the follow-up period (Table 2) demonstrated a statistically significant association for the SeLECT score (HR 1.508; 95% CI 1.183–1.923; *P* < .001). Among its individual components, only cortical lesion localization was significantly associated with unprovoked seizures (HR 3.324; 95% CI 1.432–7.714; *P* = .005). Other SeLECT score components, including initial NIHSS, large artery disease, acute symptomatic seizures after ischemic stroke, and MCA territory involvement, were not significantly associated. However, a significant association was found for NIHSS at discharge (HR 1.1; 95% CI 1.017–1.191; *P* = .018). Specifically, for NIHSS 4 to 10, the HR was 2.86 (95% CI 1.108–7.382; *P* = .03), and for NIHSS ≥ 11, the HR was 3.13 (95% CI 0.714–13.683; *P* = .13). Notably, discharge NIHSS data were unavailable for 46 patients.

**Table 2:** Univariate Cox analysis of predictors associated with unprovoked epileptic seizures.

Independent variable	HR	CI 95%	*P*	Cases with missing values
Gender – male	1.274	0.563–2.884	.562	0
Age at the time of stroke (yr)	0.992	0.963–1.023	.62	0
Length of hospitalization (d)	1.003	0.96–1.047	.897	5
Hemorrhagic transformation	1.423	0.335–6.048	.632	0
NIHSS on discharge	1.1	1.017–1.191	.018*	46
*SeLECT score parameters*				
NIHSS on admission	1.051	0.984–1.122	.139	0
Large artery disease	2.041	0.925–4.502	.077	0
Cortical localization of the lesion	3.324	1.432–7.714	.005*	0
Acute symptomatic seizure	2.577	0.605–10.979	.200	0
Affected territory of MCA	1.385	0.415–4.630	.596	0
SeLECT score	1.508	1.183–1.923	<.001*	0
*Anamnestic data*				
History of ischemic stroke	4.098	1.86–9.03	<.001*	0
Arterial hypertension	1.448	0.541–3.8	.461	5
Dyslipidemia	1.144	0.512–2.555	.743	7
Statin on admission	0.955	0.37–2.461	.924	92
Diabetes mellitus	1.971	0.883–4.401	.089	6
Atrial fibrillation	0.936	0.371–2.357	.888	7
Ischemic heart disease	0.755	0.258–2.210	.608	8
Peripheral artery disease	2.031	0.274–15.046	.488	0
Oncology disease	0.041	0–8.473	.24	7
Dementia	0.826	0.112–6.110	.851	0
Psychiatric diagnosis	1.513	0.45–5.093	.503	0
Active smoker	1.216	0.52–2.843	.653	10
Alcoholism	0.683	0.092–5.079	.710	9
*Laboratory values in serum*				
C-reactive protein	0.976	0.984–1.012	.400	0
Creatinine	0.998	0.662–1.108	.770	0
Urea	0.856	0.954–1.042	.238	0
Glucose	0.997	0.805–1.122	.884	0
Sodium	0.950	0.245–2.794	.548	0
Alanine transaminase	0.828	0.903–1.114	.761	0
Gamma-glutamyl transpeptidase	1.003	0.329–0.857	.958	0
Total cholesterol	0.531	0.146–3.447	.01*	120
High-density lipoprotein cholesterol	0.710	0.362–1.018	.671	116
Low-density lipoprotein cholesterol	0.607	0.116–0.949	.058	116
Triacylglycerol	0.332	0.984–1.012	.04*	120

CI = confidence intervals, HR = hazard ratio, NIHSS = National Institutes of Health Stroke Scale.

Among other clinical and radiological parameters (including stroke territory, occluded artery, ischemic core localization in the basal ganglia, thalamus, subcortical white matter, and deep white matter), only prior ischemic stroke was a significant predictor of vascular epilepsy (HR 4.098; 95% CI 1.86–9.03; *P* < .001). Among biochemical markers, vascular epilepsy was less frequently associated with elevated lipid profile values (total cholesterol, triglycerides), but due to the wide confidence intervals and missing values (n = 120 patients), the results were inconclusive.

**Figure 1.**
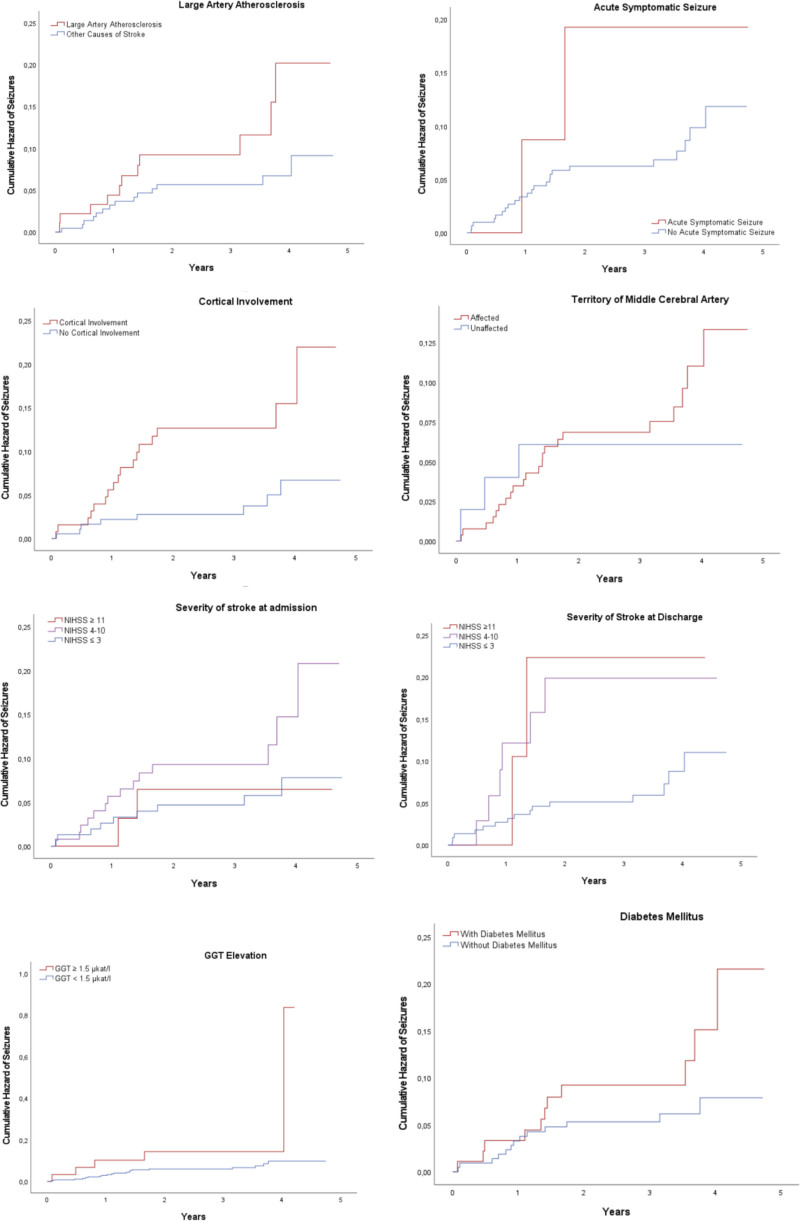
Kaplan–Meier estimates of the risk of unprovoked epileptic seizures according to individual SeLECT score parameters. NIHSS = National Institutes of Health Stroke Scale.

Kaplan–Meier risk estimation for unprovoked seizures based on SeLECT score parameters (Fig. 1) showed a statistically significant result for cortical ischemia (log-rank test; *P* = .003). Among parameters not included in the original SeLECT score, significant associations were observed for gamma-glutamyl transferase ≥ 1.5 µkat/L (*P* = .043) and the severity of neurological deficit at discharge (*P* = .037).

**Figure 2.**
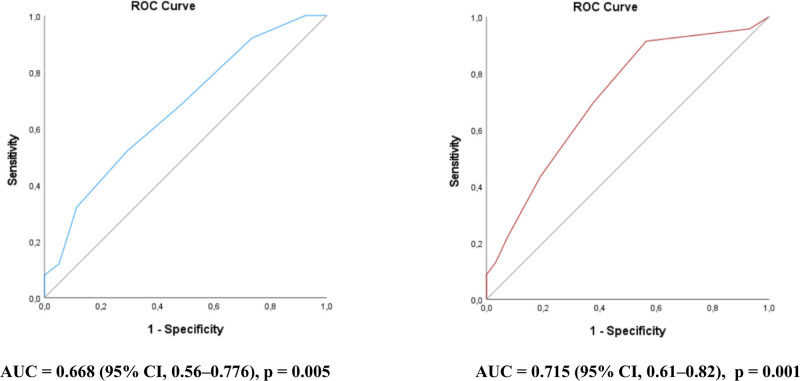
ROC curves for the original SeLECT score (left) and the modified SeLECT score calculated using discharge NIHSS (right). AUC = area under the receiver operating characteristic curve, CI = confidence intervals, NIHSS = National Institutes of Health Stroke Scale.

The cumulative risk of unprovoked seizures increased over time with higher SeLECT scores, but the growth curve showed irregularities, likely due to the low absolute number of patients with seizures. As the SeLECT score increased, specificity increased while sensitivity decreased. The optimal cutoff value for the original SeLECT score in this cohort was 4 points (Youden index 0.227), with a ROC analysis area under the receiver operating characteristic curve (AUC) of 0.668 (95% CI 0.560–0.776; *P* = .005; Fig. 2). For the modified SeLECT score, which included NIHSS at discharge instead of admission, the optimal cutoff value was 2 points (Youden index 0.348). The ROC analysis for this modified score showed a slightly higher AUC of 0.715 (95% CI 0.61–0.82; *P* = .001), though confidence intervals overlapped with those of the original model. The 1- and 5-years risk estimates for unprovoked seizures based on individual modified SeLECT scores (including NIHSS at discharge) are presented in Table 3.

**Table 3:** The risk of unprovoked epileptic seizures at 1 and 5 years after ischemic stroke for individual values of the modified SeLECT score, including the discharge NIHSS.

SeLECT score	Risk estimate at 1 yr (95% CI)	Risk estimate at 5 yr (95% CI)
0	1.6% (1.5–1.8)	2.3% (1.9–2.8)
1	2.3% (2.27–2.3)	4% (3.8–4.3)
2	3% (2.9–3.1)	5.8% (5.2–6.4)
3	4.1% (3.9–4.2)	9.6% (8.7–10.5)
4	5.5% (5.4–5.7)	14.6% (12.8–16.4)
5	7.6% (7.4–7.7)	19.2% (15.6–22.7)
6	10.3% (10.3–10.3)	33.8% (24.5–43.1)
7	13.9% (13.9–13.9)	36.9% (N/A)

CI = confidence intervals, NIHSS = National Institutes of Health Stroke Scale.

## 5. Discussion

### 5.1. Occurrence of unprovoked seizures

In this study, unprovoked seizures were observed in 7.6% of patients (n = 24), corresponding to an incidence density of 24/1000 person-years over a median follow-up period of more than 3 years. This incidence lies at the upper end of previously reported ranges in postischemic stroke patients. In a large recent Danish national cohort study (over 100,000 patients), the cumulative incidence of epilepsy after ischemic stroke was 3% after 2 years of follow-up.^[9]^ The lower cumulative incidence reported in this large study may be attributable to the incorporation of death as a competing risk, which avoids the potential overestimation inherent in traditional Kaplan–Meier methods. A Swedish study^[23]^ of 240 patients from a national registry shared similar retrospective characteristics, follow-up duration, and inclusion criteria with our cohort. With a median follow-up of 2.9 years, it reported a cumulative incidence of 5.4%, corresponding to 23 cases per 1000 person-years, which is consistent with our findings.

Since cumulative incidence increases with follow-up time, it has been shown that most unprovoked seizures occur within the first year after ischemic stroke.^[24]^ In our study, the median time to seizure was 407 days (IQR 403). Given the timing of first unprovoked seizures, the conventional 7-day threshold separating acute symptomatic seizures from vascular epilepsy after ischemic stroke^[8]^ warrants reconsideration, as it may not fully capture the underlying pathophysiological processes of epileptogenesis.

### 5.2. Risk factors and biomarkers

Among individual risk factors included in the SeLECT score, the only significant difference between the groups with vascular epilepsy and those without seizures was the presence of cortical ischemic lesions (median score of 4 vs 2 in the vascular epilepsy group, *P* = .002). This finding aligns with previous research^[23]^ and logically follows the concept of epileptogenesis after ischemic stroke. The importance of this parameter was further confirmed by Cox regression analysis (HR 3.324; 95% CI 1.432–7.714; *P* = .005) and Kaplan–Meier risk estimation (*P* = .003). Other SeLECT score parameters did not significantly differ between groups.

Among clinical characteristics, a higher median NIHSS at discharge was observed in patients with vascular epilepsy, and this parameter was a statistically significant predictor of unprovoked seizures according to Cox analysis (HR 1.1; 95% CI 1.017–1.191; *P* = .018) and Kaplan–Meier analysis (*P* = .037). Patients with vascular epilepsy were significantly less likely to have an NIHSS score below 4 (65.2% vs 85%, *P* = .035). A history of prior ischemic stroke was also a significant predictor of unprovoked seizures, according to Cox regression analysis (HR 4.098; 95% CI 1.86–9.03; *P* < .001). These results are consistent with previously identified risk factors in the literature.^[13,15]^

The predictive utility of biochemical biomarkers has generally not been strongly supported in the literature.^[25]^ However, in our study, Kaplan–Meier analysis indicated a significant association between gamma-glutamyl transferase ≥ 1.5 µkat/L and unprovoked seizures (*P* = .043). Additionally, Cox analysis suggested that lower total cholesterol and TAG levels were protective factors, although this finding should be interpreted cautiously due to wide confidence intervals and missing data. This relationship has not been well-documented in previous studies.

### 5.3. Predictive power of SeLECT score and its modifications

Consistent with 3 original SeLECT validation studies,^[15]^ our findings confirmed that patients who developed vascular epilepsy had significantly higher median SeLECT scores than those without seizures (4 vs 2; *P* = .002). Cox regression analysis showed that SeLECT score was a statistically significant risk factor for vascular epilepsy (HR 1.508; 95% CI 1.183–1.923; *P* < .001).

The cumulative risk of unprovoked seizures increased with higher SeLECT scores over time, but not for all score values, likely due to the small absolute number of patients with seizures. ROC analysis for the original SeLECT score yielded an AUC of 0.668 (95% CI 0.560–0.776; *P* = .005), slightly lower than previously reported.^[26]^ The modified SeLECT score, which included NIHSS at discharge instead of admission, showed a slightly higher AUC of 0.715 (95% CI 0.61–0.82; *P* = .001), although confidence intervals overlapped.

Although the SeLECT score has occasionally been criticized for oversimplicity,^[27]^ it remains the most widely used predictive model for vascular epilepsy after ischemic stroke due to its simplicity and well-validated prognostic properties.^[28]^ However, its predictive power can still be improved. This study demonstrated improved predictive performance by replacing admission NIHSS with discharge NIHSS in the retrospective cohort. The NIHSS at discharge reflects the final extent of brain tissue injury, which constitutes the substrate for epileptogenesis, whereas the NIHSS at admission reflects only the initial damage, which may subsequently be attenuated by the effects of acute treatment. Similar modifications have already been published, including SeLECT 2.0, which differentiates acute symptomatic seizures into short seizures (3 points) and status epilepticus (7 points), achieving higher sensitivity for identifying high-risk patients (AUC 0.84).^[17]^ SeLECT-S incorporates superficial siderosis for better risk prediction.^[16]^ SeLECT-EEG includes early electrographic biomarkers,^[18]^ achieving an agreement rate of 0.75 (95% CI: 0.71–0.80), which outperforms previous models (SeLECT 2.0: 0.71 [95% CI: 0.65–0.76]; *P* < .001). Dinç et al^[28]^ incorporated diabetes mellitus and leukoaraiosis as additional parameters, achieving an AUC of 0.955.

The SeLECT score was developed and validated as a predictive tool for late poststroke epilepsy, but it was not designed or validated to guide treatment decisions. Although current evidence does not support the use of antiseizure medication for primary prevention, as randomized trials have shown so far only limited efficacy,^[29]^ the SeLECT score may help identify high-risk subgroups for inclusion in future studies of preventive interventions.

### 5.4. Limitations of the study

The main limitations of this study stem from its retrospective nature, which naturally results in incomplete data collection and loss of patients during follow-up. Due to insufficient follow-up information, 102 patients from the Czech cohort and 198 patients from the Austrian cohort were excluded. Additionally, the Austrian cohort had limited access to data on deceased patients, leading to a lower recorded mortality rate. Another limitation was the absence of validation for the epilepsy questionnaire, which demonstrated a positive predictive value of only 47.3%. Although false positives were corrected through clinical interviews, the possibility of residual bias cannot be excluded. Seizures with non-motor manifestations, including nonconvulsive status epilepticus, may be difficult to detect through a telephone-based questionnaire, which could have resulted in false-negative responses and failure to identify the seizure. For each recorded epileptic seizure, potential causes of acute symptomatic seizures (including alcoholism) were examined to ensure that such cases were not misclassified as unprovoked seizures. However, given the retrospective nature of the study, this assessment may have been imprecise in some instances. Furthermore, patients with severe poststroke disabilities were more likely to die early or be lost to follow-up, potentially underestimating the true seizure incidence. Finally, not all patients underwent MRI, and reliance on CT scans may have led to misclassification of cortical lesion locations.

## 6. Conclusion

Vascular epilepsy was significantly associated with cortical localization of the ischemic lesion (HR 3.324; *P* = .005), higher NIHSS at discharge (HR 1.1; *P* = .018), and a history of prior stroke (HR 4.098; *P* < .001). The predictive capabilities of SeLECT score (AUC 0.668; *P* = .005) was confirmed and modifications incorporating NIHSS at discharge improved its performance (AUC 0.715; *P* = .001). The validation of the SeLECT score confirmed its usefulness in predicting vascular epilepsy. Similar to previously published modifications that enhanced the predictive capabilities of this established score, the version presented in this study, incorporating NIHSS at discharge, demonstrated higher specificity and sensitivity compared to the original model. This suggests the potential for further refinement of the model based on this score by integrating new risk factors and biomarkers that reflect the underlying pathogenetic mechanisms of epileptogenesis. Predictive models may be useful for targeted monitoring of at-risk patients and recruitment into research studies.

## Author contributions

**Conceptualization:** Petr Janský, Jaroslava Paulasová-Schwabová, Jakub Otáhal, Petr Marusič, Aleš Tomek.

**Data curation:** Petr Janský, Tereza Šrámková, Jakob Reichl, Milan S. Vosko, Lucie Šťovíčková, Anna Olšerová, Kateřina Benešová, Silvia Kmetonyová.

**Formal analysis:** Petr Janský, Tereza Šrámková, Jaroslava Paulasová-Schwabová.

**Funding acquisition:** Jakub Otáhal, Petr Marusič, Aleš Tomek.

**Investigation:** Petr Janský, Tereza Šrámková, Jakob Reichl, Milan S. Vosko, Lucie Šťovíčková, Anna Olšerová, Kateřina Benešová, Silvia Kmetonyová, Milan R. Vosko, Aleš Tomek.

**Methodology:** Petr Janský, Jaroslava Paulasová-Schwabová, Jakub Otáhal, Petr Marusič, Aleš Tomek.

**Project administration:** Petr Janský, Jakob Reichl, Lucie Šťovíčková, Anna Olšerová, Kateřina Benešová.

**Supervision:** Jakub Otáhal, Petr Marusič, Milan R. Vosko, Aleš Tomek.

**Validation:** Tereza Šrámková, Milan R. Vosko.

**Writing – original draft:** Petr Janský.

**Writing – review & editing:** Petr Janský, Jakob Reichl, Milan S. Vosko, Jakub Otáhal, Petr Marusič, Milan R. Vosko, Aleš Tomek.
